# Mitochondrial DNA in plasma and long-term physical recovery of critically ill patients: an observational study

**DOI:** 10.1186/s40635-024-00690-z

**Published:** 2024-11-06

**Authors:** Maryory Galvis-Pedraza, Lise F. E. Beumeler, Elisabeth C. van der Slikke, E. Christiaan Boerma, Tim van Zutphen

**Affiliations:** 1 Department of Sustainable Health, Faculty Campus Fryslân, University of Groningen, Leeuwarden, The Netherlands; 2 Department of Intensive Care, Medical Centre Leeuwarden, Leeuwarden, The Netherlands; 3 Research Group Digital Innovation in Healthcare and Social Work, NHL Stenden University of Applied Sciences, Leeuwarden, The Netherlands; 4 Department of Clinical Pharmacy and Pharmacology, University of Groningen, University Medical Center Groningen, Groningen, The Netherlands

**Keywords:** Mitochondrial DNA, Mitochondrial dysfunction, Critical illness, Long-term recovery, PICS

## Abstract

**Background:**

Post-intensive care syndrome (PICS) poses a notable public health concern, with survivors of critical illness experiencing long-term physical, psychological, and cognitive challenges. Mitochondrial dysfunction has gained attention for its potential involvement in PICS. However, the long-term impact of mitochondrial status on patient recovery remains uncertain. A single-centre retrospective analysis was conducted in Leeuwarden, the Netherlands, between May and November 2019, within a mixed ICU survivor cohort. Patients were assessed for mitochondrial markers (mtDNA damage represented by the presence of mtDNA fragmentation and mitochondrial DNA levels evaluated by the ratio of mtDNA and nuclear DNA), clinical factors, and long-term outcomes measured by the physical functioning (PF) domain of health-related quality of life.

**Results:**

A total of 43 patients were included in this study divided into recovery and non-recovery groups based on age-adjusted PF scores at 12 months post-ICU. Nineteen patients scored below these thresholds. No significant differences in mitochondrial markers between groups were identified. Furthermore, no significant correlations were found between mtDNA levels and mtDNA damage at baseline and 12 months with PF scores. However, mtDNA levels decreased over time in the recovery (*p*-value <  < 0.01) and non-recovery groups (*p*-value < 0.01).

**Conclusion:**

No significant correlation was found between mitochondrial markers and physical functioning scores. This study underscores the multifactorial nature of PICS and the need for a comprehensive understanding of its metabolic and cellular components. While mitochondrial markers may play a role in PICS, they operate within a framework influenced by various factors. This exploratory study serves as a foundation for future investigations aimed at developing targeted interventions to enhance the quality of life for ICU survivors grappling with PICS.

**Supplementary Information:**

The online version contains supplementary material available at 10.1186/s40635-024-00690-z.

## Background

Multiple organ dysfunction syndrome (MODS) is a common complication in critical illness and a leading cause of mortality in these patients. Mitochondrial dysfunction has been proposed to represent a pivotal factor in MODS multifactorial aetiology [1]. Under physiological conditions, mitochondria serve as cellular powerhouses, essential for a wide range of energy-demanding processes. During critical illness, the overwhelming formation of reactive oxygen species (ROS) may induce mitochondrial dysfunction and precipitate a cascade of events, culminating in cell function shutdown and even programmed cell death [2, 3]. As such, current evidence emphasizes the central role of mitochondrial dysfunction in the development of MODS [4] during acute illness, both in the context of sepsis and non-sepsis conditions [5, 6]. Muscle mitochondria are particularly directly associated with physical function [7]. However, the role of the restoration of mitochondrial function in the recovery phase of critically ill patients remains elusive.

The survivors of critical illness frequently suffer from physical, psychological, and cognitive challenges post-intensive care unit (ICU) discharge, collectively known as PICS. Today, PICS is increasingly recognized for its significant impact on the quality of life of those patients [8]. Recently, mitochondrial dysfunction has gained attention as underlying mechanism in ICU-acquired physical impairments [9]. This prompts consideration for a role for mitochondrial function beyond the phase of acute critical illness, potentially relevant in the phase of long-term recovery as well. However, the relationship between mitochondrial markers and long-term quality of life after critical illness, particularly physical functioning (PF), remains unexplored [10].

To address this knowledge gap, the current exploratory study made use of our recently established Frisian aftercare cohort [11] and described markers of mitochondrial DNA levels (evaluated by the ratio of mtDNA and nuclear DNA) and mitochondrial damage (mtDNA damage represented by the presence of mtDNA fragmentation) at baseline and 12 months post-ICU. Further, the relationship between mitochondrial markers and long-term outcomes of PF was explored.

## Methods

### Study design

This is a retrospective, single-centre, observational study with an analytical scope nested in a Frisian ICU survivors cohort, as established by Beumeler et al., [11]. Data were collected in a tertiary teaching hospital with a mixed ICU, located in Leeuwarden, the Netherlands. The participants were adult patients admitted to the ICU between May and November 2019 with a length of stay (LOS) of ≥ 48 h. Only patients for whom plasma mitochondrial measurements were successfully assessed were included in the analysis.

### Quantification of mitochondrial DNA levels and mitochondrial DNA damage using real-time qPCR

We employed real-time quantitative polymerase chain reaction (qPCR) to assess the mtDNA extracted from plasma samples collected from venous blood, as described previously by van der Slikke et al., [10], at baseline (< 72 h after admission) and 12 months post-ICU. To estimate mtDNA levels, cycle threshold (CT) values of NADH dehydrogenase 1 (ND1) and beta-2 microglobulin (β2M) were evaluated, with the ND1/β2M ratio reflecting the relative abundance of the mitochondrial genome. For amplification of the DNA the following protocol was used: 95 °C for 2 min, and 40 cycles of 95 °C for 15 s and 61 °C for 60 s. All reactions were carried out in duplicate and CT values were averaged. A standard curve was used to determine the efficiency, linear range, and reproducibility of the qPCR assay. The difference in the ct value between ND1 and B2M was used to quantify the relative abundance of the mitochondrial genome. MtDNA damage was assessed by quantifying long-range PCR, where the ratio between a long and short mtDNA fragment was calculated [10]. TaKaRa LA Taq DNA polymerase kit (Takarabio, Kusatsu, Japan) was used for the long-range PCR. A 10 kb mtDNA template, stretching from ND5 to ND1, representing more than two-third of the mitochondrial genome, was amplified by long-range PCR (Biorad, California, USA), while the reference consisted of a short mtDNA fragment of approximately 200bp (D-loop; a regulative region). 5 μl of DNA was used. For the amplification of the long 10 kb mtDNA part, the following thermal profile was used: To amplify the long-range DNA, 94 °C for 1 min, followed by 18 cycles of 15 s at 94 °C and 12 min at 64 °C was used, followed by 10 min at 72 °C. The short fragment was amplified using: 95 °C for 2 min, followed by 40 cycles of 95 °C for 15 s and 61 °C for one min. Both PCR products were run on a 1% agarose gel (45 min, 100 V). The intensity of the bands on the gel was analysed using Image Lab (Biorad, California, USA). The ratio of the intensity of the short stable fragment to the long unstable fragment was calculated to quantify mtDNA damage. A higher ratio reflects more mtDNA damage.

### Physical functioning and group allocation

Evaluation of the PF domain of health-related quality of life (HRQoL) was performed by the use of a validated Dutch equivalent of the 36-item Short Form Health Survey, the Research and Development-36 (RAND-36) questionnaire[12]; administered at baseline (to assess pre-ICU physical functioning) and 12 months after discharge from ICU. In cases where patients were unable to fill in the questionnaires at baseline, proxies were asked to perform this task[11]. Patients were then categorized into recovery (R) and non-recovery (NR) groups based on their 12-month PF score. To establish appropriate cut-off values, we used age-specific thresholds in RAND-36 PF domain score obtained from Table 5 in the manual by van der Zee et al., [12].

### Demographic factors and clinical characteristics

The following demographic factors and clinical characteristics were collected: age on admission, gender, LOS ICU, LOS hospitalization (LOS hos), body mass index (BMI), type of admission, sepsis, need for cardiopulmonary resuscitation (CRP) and Clinical Frailty Scale (CFS) score at admission. The medical comorbidities such as the presence of malignancy, diabetes, chronic obstructive pulmonary disease (COPD), history of cerebrovascular accident (CVA) and chronic kidney disease (CKD) were included following the data dictionary of the National Intensive Care Evaluation [13]. Additionally, information on indices of severity of illness during ICU stay was recorded, including the need for renal replacement therapy (RRT), the need for intubation, the number of days on mechanical ventilation and the Acute Physiology and Chronic Health Evaluation (APACHE III) score.

### Statistical analysis

The descriptive statistics were applied to characterize the population, combined with a Shapiro–Wilk test was applied to numerical variables to assess the data for normality. Consequently, scale variables were summarized as median and interquartile range, while categorical variables were described as proportions. A *p*-value of less than 0.05 was considered the threshold for statistical significance.

Differences between the recovery (R) and non-recovery (NR) groups were evaluated using a Wilcoxon Rank Sum Test for scale variables. Fisher’s Exact Test was employed to evaluate contingency tables for categorical variables such as gender, admission type, and comorbidities. Correlation coefficients were calculated for the relationship between mitochondrial markers and the scores from PF evaluated at baseline and 12 months, reported as Spearman’s Rho (r_s_). Additionally, the correlation between clinical variables and mitochondrial markers and PF was evaluated. While the sample size may limit statistical power, the primary goal was identifying trends and generating hypotheses.

Due to the exploratory nature of this study, a visual overview was created using boxplots to identify potential patterns, for which PF scores were categorized into quartiles and mitochondrial markers were evaluated in the PF quartiles.

All statistical analyses were performed using the statistical software package R version 4.3.1 (Beagle Scouts). Tables for the final report were made using Microsoft Excel (Microsoft Office Professional Plus 2019). The results of this study were reported using the Strengthening the Reporting of Observational Studies (STROBE) checklist [14].

## Results

### Participants

**Fig. 1:**
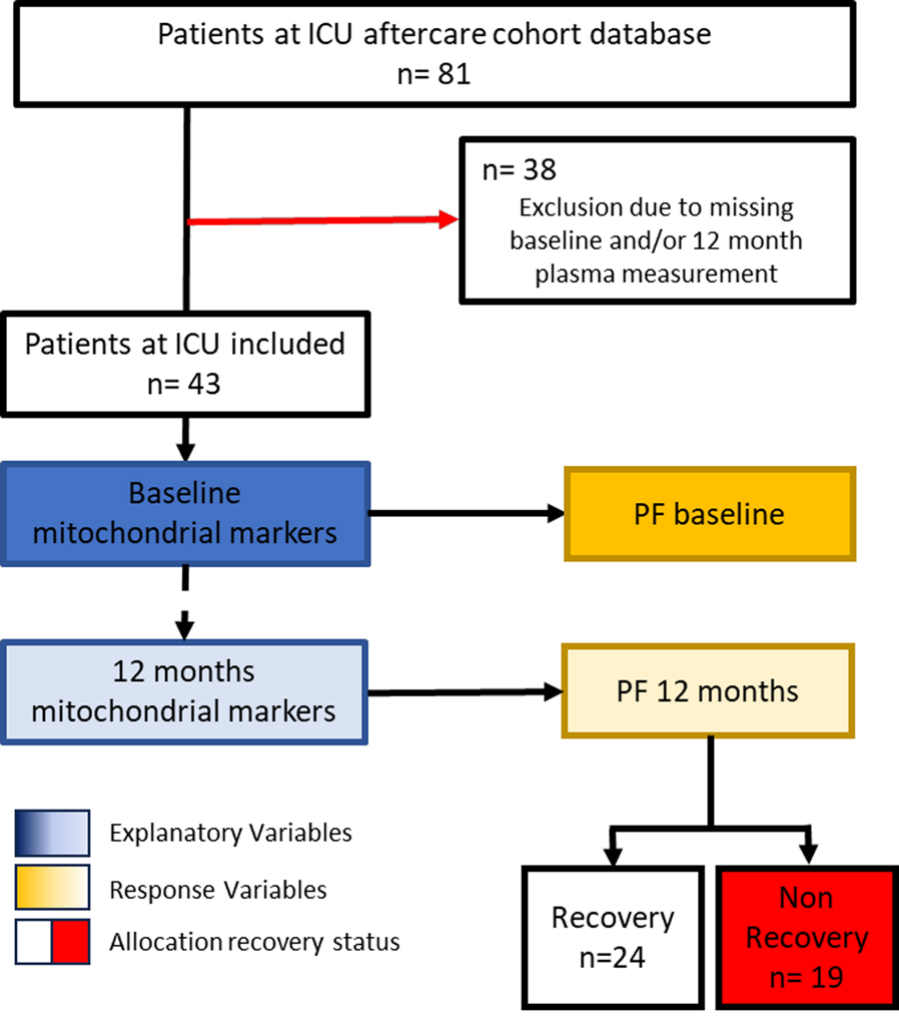
Flowchart of study inclusion and group allocation

Out of 81 available patients, 43 had records of plasma mitochondrial markers at baseline and 12 months and were included in the study (Fig. 1).

### Descriptive data

Most of the patients were of male (79%) and were severely ill (APACHE III: 83 [60–104]) (Table 1). The median age was 64 [53–73]. In total, 56% (*n* = 24) of patients were allocated to the R-group. Besides a significantly higher occurrence of COPD and more days on mechanical ventilation (MV) (*p* = 0.02 and *p* = 0.05, respectively), there were no significant differences between the R and NR-group (Table 1).

**Table 1:** Demographic factors and clinical characteristics of ICU patients and by recovery status at 12 months

	All [*n* = 43]	Recovery [*n* = 24]	Non-recovery [*n* = 19]	*p*-value
Demographics				
Age (years)	64 [53–73]	62 [49–75]	65 [57–72]	0.34
Gender (% men)	34 (79)	21 (88)	13 (68)	0.13
Body Mass Index (kg/m^2^)	28 [24–32]	27 [24–31]	29[25–32]	0.17
Clinical Frailty Score	3 [2–4]	3 [2, 3]	4 [2–5]	0.19
Comorbidities, *n*(%)				
Malignancy	3 (7)	3 (13)	0 (0)	0.11
Diabetes	7 (16)	2 (8)	5 (26)	0.11
COPD	4 (9)	0 (0)	4 (21)	0.02*
CVA	3 (7)	2 (8)	1 (5)	0.69
CKD	3 (7)	2 (8)	1 (5)	0.69
Admission type, *n*(%)				0.63
Medical	23 (54)	13 (54)	10 (53)	
Surgical, acute	11 (26)	5 (21)	6 (32)	
Surgical, elective	9 (21)	6 (25)	3 (16)	
Reason for admission, *n*(%)				0.52
CPR	10 (23)	6 (25)	4 (21)	
Sepsis	8 (19)	3 (13)	5 (26)	
Other	25 (58)	15 (60)	10 (53)	
ICU characteristics				
APACHE III score	83 [60–104]	85 [68–103]	80 [56–98]	0.35
Intubation, *n*(%)	39 (90.7)	22 (91.7)	17 (89.5)	0.81
MV, days	5 [1–6]	5 [1–4]	7 [3–7]	0.05*
RRT, *n*(%)	8 (18.6)	4 (16.7)	4 (21.1)	0.71
LOS ICU, days	8 [4–10]	7 [3–8]	10 [5–13]	0.10

Data are presented as median [IQR] unless stated otherwise

APACHE, acute physiology and chronic health evaluation; CKD, chronic kidney disease; COPD, chronic obstructive pulmonary disease; CPR, cardiopulmonary resuscitation; CVA, cerebrovascular accident; LOS ICU, length of stay intensive care unit; MV, mechanical ventilation; RRT, renal replacement therapy; SOFA, simplified acute physiology score

*Statistically significant. Group alignment (recovery/non-recovery) based on age-adjusted threshold for the RAND-36 physical functioning score at 12 months: 91.7 [18–24y], 89.5 [25–34y], 90.0 [35–44y], 79.9 [45–54y], 72.7 [55–64y], 66.7 [65–75y], 56.0 [75–85y], 60.0 [85 + y]

Descriptive data on mitochondrial markers are shown in Table 2. There seems to be a general decline in mtDNA levels over time and an absolute increase of mtDNA damage specifically in the NR-group between baseline and 12 months post-ICU. However, there were no statistically significant differences observed in any of the variables when comparing the R and NR-group. Furthermore, the spread of IQRs in mtDNA damage indicated the presence of heterogeneity in the results.

**Table 2:** Baseline and 12-month follow-up characteristics of mitochondrial markers in patients grouped by physical recovery status

	All	Recovery	Non-recovery	*p*-value
	[*n* = 43]	[*n* = 24]	[*n* = 19]	
Mitochondrial markers at baseline				
mtDNA levels	0.71 (0.05)	0.71 (0.04)	0.71 (0.05)	0.68
mtDNA damage	26.18 [11.18–42.88]	28.60 [14.02–55.87]	20.15 [9.15–33.39]	0.21
Mitochondrial markers at 12 months				
mtDNA levels	0.65 (0.06)	0.64 (0.04)	0.65 (0.07)	0.80
mtDNA damage	28.52 [12.90–46.12]	28.06 [20.33–51.86]	28.61 [9.96–41.31]	0.26
Change in mitochondrial markers in time				
∆mtDNA	− 0.06 (0.06)	− 0.06 (0.06)	− 0.06 (0.07)	0.94
∆mtDNA damage	0.72 [− 16.79 to 22.84]	0.06 [− 15.05 to 16.07]	0.72 [− 16.79 to 25.45]	0.89

Data are presented as mean (SD) for mtDNA levels and median [IQR] for mtDNA Damage

mtDNA levels, represented by ND1/B2M-ratio; mtDNA Damage, Mitochondrial DNA damage; ∆mtDNA levels, Change in mt DNA levels from baseline to 12 months; ∆mtDNA damage, Change in mitochondrial DNA damage from baseline to 12 months

A Wilcoxon signed-rank test was applied to evaluate differences between mitochondrial markers at baseline and after 12 months for the whole group. The findings indicate a significant difference in mtDNA levels between baseline and 12 months (*p*-value <  < 0.01), suggesting a notable decrease in mtDNA levels over the year. Further analysis showed a significant difference in mtDNA levels for both the recovery group (*p*-value <  < 0.01) and the non-recovery group (*p*-value < 0.01). However, there was no significant difference in mtDNA damage between baseline and 12 months for the whole group. Similarly, there was no significant difference in mtDNA damage for both the recovery group and the non-recovery group (all p-values > 0.77).

### Correlations with physical functioning

No significant correlations were found between mtDNA levels and the PF domain score at baseline and 12 months (r_s_ baseline: − 0.21, r_s_ baseline-12m: -0.09, r_s_ 12m: − 0.11) (see ESM 1, Table S1). In addition, the relationship between mtDNA damage and physical function recovery status indicated varying degrees of heterogeneity and no significant associations were identified (r_s_ baseline: 0.02, r_s_ baseline-12m: 0.22, r_s_ 12m: 0.08). A similar lack of significant correlations was found when investigating the relationship between PF at baseline and 12m to ∆mtDNA levels and ∆mtDNA damage (see ESM1, Table S2).

Significant correlations were observed between some clinical characteristics and mitochondrial markers (see ESM, Table S3). In the recovery group, a strong positive correlation (*r* = 0.63, *p* = 0.01) was found between CFS and mtDNA damage at 12 months. In the non-recovery group, significant correlations were found between SAPS and mtDNA damage at baseline (*r* = 0.515, *p* = 0.024), and between APACHEIII scores and mtDNA damage at baseline (*r* = 0.625, *p* = 0.004). No significant correlations were found between other factors and mtDNA levels, mtDNA damage, or changes in these markers over time (∆).

**Fig. 2:**
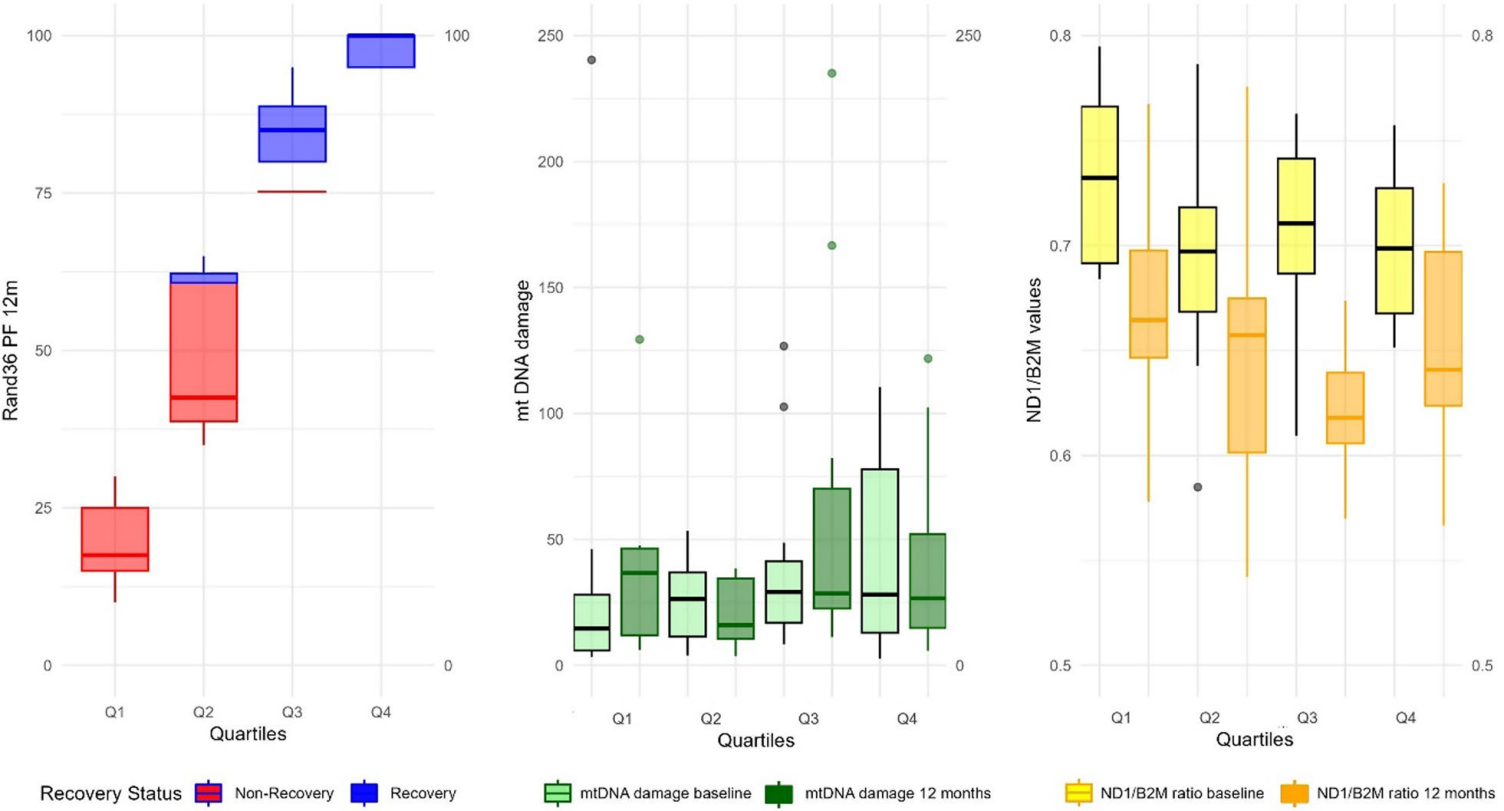
Distribution of mitochondrial values and physical functioning recovery status at 12 months post-ICU, in physical recovery quartiles. Boxplots display data with whiskers extending to the smallest and largest values within 1.5 times the interquartile range (IQR) from the first (Q1) and third quartiles (Q3), following Tukey’s method. Outliers outside this range are shown as individual dots. The ND1/B2M ratio refers to the mitochondrial DNA levels and Q1-Q4 represent physical functioning quartiles

Figure 2 provides more detailed information on the distribution of physical functioning scores and mitochondrial markers at 12 months post-ICU. While initially patients were allocated to the R or NR-group, there seemed to be some overlap in Q2 and Q3 of the PF distribution. To substantiate the findings in the previously explored correlations, no visual relations could be found between the PF-quartiles, mtDNA levels and mtDNA damage scores at baseline or 12 months post-ICU. However, mtDNA levels values seemed higher at baseline compared to 12 months in all quartiles.

## Discussion

This study aimed to explore a possible relation between mitochondrial markers and long term PF recovery status in patients after critical illness. Specifically, we investigated whether plasma mtDNA levels and mtDNA damage are linked to PF in survivors. The findings revealed heterogeneity in these markers, with no significant correlation to PF at baseline or 12 months post-ICU. These results suggest that the use of these plasma mitochondrial markers may not be feasible for differentiating between PF recovery and non-recovery after critical illness.

Nevertheless, these observations do not explore the role of mitochondrial function in ICU recovery to the full extent. It is worth mentioning that similar studies conducted in the Netherlands investigating the use of mtDNA levels and mtDNA damage as biomarkers also did not yield consistent effects [15, 16]. Additionally, the studies concentrating on the inflammatory response with and without sepsis have pointed to elevated levels of mtDNA and other cellular markers as a primary risk factor for increased mortality, particularly during cardiac arrest [17], while others have identified an increase in damage-associated molecular patterns (DAMPs) produced by mitochondrial lesions [18, 19]. Furthermore, a study on sepsis patients found higher levels of oxidated DNA, RNA, and lipid peroxidation compared to controls, with increased plasma mtDNA and mtDNA damage linked to higher long-term mortality [10]. These findings reinforce the connection between mitochondrial damage and severe outcomes in critical illness.

While the relationship between mitochondrial markers and PF recovery remains unclear, this study revealed a few other significant correlations: in the recovery group, a strong positive correlation was observed between CFS and mtDNA damage at 12 months (*r* = 0.63, *p* = 0.01), suggesting that higher clinical frailty might be linked to increased mitochondrial damage over time. In the non-recovery group, significant correlations were found between SAPS and mtDNA damage at baseline (*r* = 0.515, *p* = 0.024), and between APACHEIII scores and mtDNA damage at baseline (*r* = 0.625, *p* = 0.004), suggesting a relationship between disease severity and mitochondrial dysfunction. These results align with studies that show mitochondrial dysfunction, including mtDNA damage, are associated with conditions such as sarcopenia, aging, and metabolic dysfunctions, which are closely related to disease severity scores [20, 21]. These findings need to be interpreted with some caution, given the exploratory design of the study.

Although these correlations provide some information on the relationship between mitochondrial markers and critical illness, the impact of cardiac conditions [15, 16], systemic inflammatory response [17], and acute liver failure [18], among others, during ICU admission and the recovery phase must be taken into account. Also, it is commonly known that comorbidities at ICU admission have a negative effect on long term recovery; mitochondrial functioning at baseline could be relevant in the development and progression of PICS [7, 22].

In this study, mtDNA levels decreased over time in most patients. Despite this trend, no correlation or association with PF scores was observed. Still, previous research has discussed mtDNA as a potential biomarker for physical function recovery. For instance, it has been suggested as a predictor for physical recovery and outcomes in critically ill patients, helping to monitor response to treatment, and identify those at higher risk for chronic conditions post-ICU stay [23, 24]. In another study, a small significant difference was found in plasma mtDNA levels between patients with a good and a poor outcome at day 1, but this difference diminished at day 7 and day 14 post admission [25]. Finally, our study did not find mtDNA markers to be a predictor of MV, APACHE or other outcome associated with severity of critical illness in septic patients, unlike findings in other studies [10, 25]. This emphasizes the need for more suitable biomarkers for long term physical recovery.

To our knowledge, this is the first study investigating markers for mitochondrial damage up to 12 months post-ICU. Nevertheless, caution should be applied in generalizing our results due to the retrospective design and reliance on patient-reported outcomes, and the potential influence of preexisting disabilities. Non-recovery status is not corrected for these disabilities in our approach. Additionally, baseline levels of mt markers may have been acutely affected by the critical illness itself within the time window for collecting the blood samples. While the RAND-36 is a widely validated tool for assessing physical functioning, responses in ICU survivors may be influenced by stress. Its reliability has been well-established across diverse patient populations in the Netherlands [26, 27]. The evidence shows moderate correlations between patient-reported outcomes and objective measures of physical function, supporting the use of the RAND-36 in similar contexts [28, 29]. Also, during to the COVID-19 pandemic, it was not possible to collect plasma samples for all patients, which may have limited our ability to detect potentially clinically relevant associations. Furthermore, due to the exploratory nature of this paper and its limited sample size no multivariate analyses or corrections have been applied. Still, our results emphasize the multifactorial nature of PICS.

## Conclusions

This study has provided insights into the distribution of plasma markers of mitochondrial dysfunction at baseline and 12 months post-ICU. No clear correlation was found between these markers and physical functioning. Further investigations are warranted to identify and target specific factors influencing ICU survivors’ outcomes, ultimately improving their overall recovery and quality of life.

## Supplementary Information

Additional file 1.

## Data Availability

The datasets generated and/or analyzed during the current study are available from the Zenodo database (10.5281/zenodo.6656128), as referenced in Beumeler LFE et al. [11]. The data includes patient demographics and RAND-36 survey results. The mitochondrial function data, which are not included in the Zenodo repository, can be provided by the corresponding author upon reasonable request.

## Abbreviations

PICS: Post-intensive care syndrome
mtDNA: Mitochondrial DNA
ICU: Intensive care unit
PF: Physical functioning
LOS: Length of stay
qPCR: Quantitative polymerase chain reaction
ND1: NADH dehydrogenase 1
β2M: Beta-2 microglobulin
HRQoL: Health-related quality of life
RAND-36: Research and Development 36-item Short Form Health Survey
CFS: Clinical frailty scale
COPD: Chronic obstructive pulmonary disease
CVA: Cerebrovascular accident
CKD: Chronic kidney disease
APACHE III: Acute physiology and chronic health evaluation III
RRT: Renal replacement therapy
MV: Mechanical ventilation
MODS: Multiple organ dysfunction syndrome
ROS: Reactive oxygen species
DAMPs: Damage-associated molecular patterns
