# Supplementary material for: Mitochondrial DNA in plasma and long-term physical recovery of critically ill patients: an observational study
Source: Intensive Care Med Exp. 2024 Nov 6;12:99. doi: 10.1186/s40635-024-00690-z (PMC11541963; doi:10.1186/s40635-024-00690-z)
Supplement: Supplementary file 1 — Additional file 1. [file 40635_2024_690_MOESM1_ESM.docx]

# **Electronic supplementary material 1**

***Table 1. Correlation between mitochondrial values and PF*.**

| **mtDNA levels (ND1/B2M ratio)** | **PF** | **S. Rho** | **p-value** |
| --- | --- | --- | --- |
| Baseline | Baseline | -0.21 | 0.178 |
|  | 12m | -0.09 | 0.564 |
| 12m | 12m | -0.11 | 0.491 |
| **mtDNA damage** | **PF** | **S. Rho** | **p-value** |
| Baseline | Baseline | -0.02 | 0.916 |
|  | 12m | 0.22 | 0.165 |
| 12m | 12m | 0.08 | 0.624 |

m: months; PF: Physical Function; S.Rho: Spearman’s Rho. The correlation analysis between mtDNA levels (ND1/B2M ratio) and mtDNA damage with PF were made at two time points. mtDNA markers at baseline were crossed against both, PF at baseline and PF after 12m. mtDNA markers at 12m were just crossed against PF score at 12m. S. Rho values and corresponding p-values are shown for each correlation. The analysis revealed no significant correlations between mtDNA levels or mtDNA damage markers and PF, neither at baseline nor at 12 months.

## ***Table 2. Correlations between ∆mitochondrial values and PF.***

| ***∆*mtDNA levels (ND1/B2M ratio)** | | |
| --- | --- | --- |
| **PF** | **S. Rho** | **p-value** |
| Baseline | 0.05 | 0.744 |
| 12m | -0.01 | 0.972 |
| *∆***mtDNA damage** | | |
| **PF** | **S. Rho** | **p-value** |
| Baseline | -0.18 | 0.252 |
| 12m | -0.09 | 0.557 |

m: months; PF: Physical Function; S.Rho: Spearman’s Rho. The correlation analysis between ∆mtDNA levels (ND1/B2M ratio) and ∆mtDNA damage with PF were made at two time points, baseline and 12m. S. Rho values and corresponding p-values are shown for each correlation. The analysis revealed no significant correlations between ∆mtDNA levels or∆ mtDNA damage markers and PF, neither at baseline nor at 12 months.

***Table 3. Correlations between mitochondrial markers and clinical variables***

**3.1 Mitochondrial Markers from Recovery Group Data Table and p-values**

| Demographic/  Clinical Value | mtDNA damage baseline | p-value | mtDNA damage 12m | p-value | ND1_B2M ratio baseline | p-value | ND1_B2M ratio 12m | p-value | delta mtDNA damage | p-value | delta ND1_B2M ratio | p-value |
| --- | --- | --- | --- | --- | --- | --- | --- | --- | --- | --- | --- | --- |
| Age admission | -0.23 | 0.35 | 0.16 | 0.50 | -0.06 | 0.80 | 0.06 | 0.80 | 0.24 | 0.35 | -0.04 | 0.90 |
| LOS_IC | 0.15 | 0.60 | -0.12 | 0.65 | 0.43 | 0.07 | 0.03 | 0.90 | -0.15 | 0.60 | -0.25 | 0.40 |
| LOS_hos | -0.03 | 0.90 | 0.20 | 0.40 | 0.14 | 0.60 | 0.02 | 0.95 | 0.29 | 0.30 | -0.08 | 0.75 |
| Admission Type (Medical) | 0.38 | 0.07 | 0.01 | 0.98 | -0.28 | 0.18 | -0.13 | 0.56 | -0.30 | 0.16 | 0.04 | 0.85 |
| Admission Type (Elective surgical) | -0.13 | 0.56 | 0.39 | 0.06 | 0.23 | 0.28 | 0.19 | 0.39 | 0.41 | 0.05 | -0.01 | 0.97 |
| Admission Type (Acute surgical) | -0.32 | 0.13 | -0.38 | 0.07 | 0.11 | 0.61 | -0.03 | 0.90 | -0.04 | 0.85 | -0.04 | 0.85 |
| Sepsis | 0.14 | 0.60 | 0.14 | 0.60 | 0.24 | 0.40 | -0.44 | 0.12 | -0.20 | 0.45 | -0.44 | 0.12 |
| CPR | -0.08 | 0.75 | -0.18 | 0.50 | -0.39 | 0.15 | -0.18 | 0.50 | -0.13 | 0.65 | 0.00 | 1.00 |
| RRT_ICU | 0.07 | 0.80 | -0.16 | 0.55 | 0.13 | 0.65 | 0.01 | 0.95 | -0.13 | 0.65 | -0.07 | 0.80 |
| APACHEIII | 0.18 | 0.55 | 0.00 | 1.00 | -0.11 | 0.70 | 0.13 | 0.65 | -0.07 | 0.80 | 0.12 | 0.70 |
| SAPS | 0.32 | 0.13 | 0.06 | 0.80 | -0.02 | 0.95 | 0.12 | 0.70 | -0.26 | 0.26 | 0.08 | 0.75 |
| SOFA | -0.04 | 0.85 | -0.19 | 0.48 | 0.12 | 0.70 | 0.09 | 0.75 | -0.02 | 0.95 | 0.04 | 0.85 |
| CFS | 0.28 | 0.20 | 0.63 | 0.01 | 0.24 | 0.40 | -0.12 | 0.70 | 0.18 | 0.50 | -0.19 | 0.48 |

**3.2 Mitochondrial Markers from Non-Recovery Group Data Table**

| Demographic/Clinical Value | mtDNA damage baseline | p-value | mtDNA damage 12 months | p-value | ND1_B2M ratio baseline | p-value | ND1_B2M ratio 12 months | p-value | delta mtDNA damage | p-value | delta ND1_B2M ratio | p-value |
| --- | --- | --- | --- | --- | --- | --- | --- | --- | --- | --- | --- | --- |
| Age admission | 0.03 | 0.89 | 0.16 | 0.52 | -0.06 | 0.80 | 0.06 | 0.80 | 0.24 | 0.35 | -0.04 | 0.90 |
| LOS_IC | 0.39 | 0.10 | -0.12 | 0.65 | 0.43 | 0.07 | 0.03 | 0.90 | -0.15 | 0.60 | -0.25 | 0.40 |
| LOS_hos | 0.25 | 0.30 | 0.20 | 0.40 | 0.14 | 0.60 | 0.02 | 0.95 | 0.29 | 0.30 | -0.08 | 0.75 |
| Admission Type (Medical) | 0.14 | 0.58 | 0.33 | 0.17 | 0.10 | 0.70 | 0.12 | 0.64 | -0.30 | 0.16 | 0.04 | 0.85 |
| Admission Type (Elective surgical) | 0.08 | 0.74 | -0.04 | 0.87 | 0.10 | 0.67 | -0.02 | 0.93 | 0.12 | 0.61 | -0.12 | 0.61 |
| Admission Type (Acute surgical) | -0.29 | 0.23 | -0.40 | 0.09 | -0.26 | 0.28 | -0.13 | 0.59 | -0.11 | 0.67 | 0.16 | 0.52 |
| Sepsis | -0.26 | 0.28 | 0.14 | 0.60 | 0.24 | 0.40 | -0.44 | 0.12 | -0.20 | 0.45 | -0.44 | 0.12 |
| CPR | 0.17 | 0.50 | -0.18 | 0.50 | -0.39 | 0.15 | -0.18 | 0.50 | -0.13 | 0.65 | 0.00 | 1.00 |
| RRT_ICU | 0.12 | 0.63 | -0.16 | 0.55 | 0.13 | 0.65 | 0.01 | 0.95 | -0.13 | 0.65 | -0.07 | 0.80 |
| APACHEIII | 0.63 | 0.004 | 0.00 | 1.00 | -0.11 | 0.70 | 0.13 | 0.65 | -0.07 | 0.80 | 0.12 | 0.70 |
| SAPS | 0.52 | 0.02 | 0.06 | 0.80 | -0.02 | 0.95 | 0.12 | 0.70 | -0.26 | 0.26 | 0.08 | 0.75 |
| SOFA | 0.35 | 0.15 | -0.19 | 0.48 | 0.12 | 0.70 | 0.09 | 0.15 | 0.01 | -0.17 | -0.35 | 0.14 |
| CFS | -0.43 | 0.10 | -0.07 | 0.81 | 0.21 | 0.44 | -0.12 | 0.66 | 0.26 | 0.33 | -0.29 | 0.28 |
